# The MST/Hippo Pathway and Cell Death: A Non-Canonical Affair

**DOI:** 10.3390/genes7060028

**Published:** 2016-06-17

**Authors:** Emma Fallahi, Niamh A. O’Driscoll, David Matallanas

**Affiliations:** 1 Systems Biology Ireland, University College Dublin, Belfield, Dublin 4, Ireland; emma.fallahi---sichani@ucdconnect.ie (E.F.), niamh.o-driscoll@ucdconnect.ie, (N.A.O.); 2 School of Medicine and Medical Science, University College Dublin, Belfield, Dublin 4, Ireland

**Keywords:** Hippo pathway, signalling network, apoptosis, cancer, diabetes, neurodegenerative disease, YAP, MST1/2, LAST1/2, RASSF1A

## Abstract

The MST/Hippo signalling pathway was first described over a decade ago in *Drosophila melanogaster* and the core of the pathway is evolutionary conserved in mammals. The mammalian MST/Hippo pathway regulates organ size, cell proliferation and cell death. In addition, it has been shown to play a central role in the regulation of cellular homeostasis and it is commonly deregulated in human tumours. The delineation of the canonical pathway resembles the behaviour of the Hippo pathway in the fly where the activation of the core kinases of the pathway prevents the proliferative signal mediated by the key effector of the pathway YAP. Nevertheless, several lines of evidence support the idea that the mammalian MST/Hippo pathway has acquired new features during evolution, including different regulators and effectors, crosstalk with other essential signalling pathways involved in cellular homeostasis and the ability to actively trigger cell death. Here we describe the current knowledge of the mechanisms that mediate MST/Hippo dependent cell death, especially apoptosis. We include evidence for the existence of complex signalling networks where the core proteins of the pathway play a central role in controlling the balance between survival and cell death. Finally, we discuss the possible involvement of these signalling networks in several human diseases such as cancer, diabetes and neurodegenerative disorders.

## 1. Introduction

The Hippo (Hpo) pathway was originally described in *Drosophila melanogaster* over a decade ago [1]. The work from different groups demonstrated that the core of this pathway is conserved throughout evolution [2]. Since the characterisation of the mammalian pathway in 2007 [3,4] there has been increasing interest in this network due to its central role in the regulation of biological functions such as cell proliferation, survival, mechanotransduction, organogenesis, stem cell self-renewal and organ size [1,5,6]. The physiological functions of the mammalian Hippo pathway caught the attention of the research community and evidence that several members of the pathway are involved in cancer development sparked further interest in this pathway [2,7,8]. The summary of this work has led to the description of the so called canonical Hippo pathway which includes proteins that were shown to be part of the pathway in genetic studies performed in the fly [1,9]. By analogy to *Drosophila*, the core of the canonical mammalian pathway is formed by the homologues of the fly proteins; the serine/threonine kinases MST1/2 (Hippo) and LATS1/2 (Warts), the scaffold Salvador (Sav), the adaptor protein MOB (Mats), the transcriptional co-activators YAP1/2 and TAZ (Yki) and the family of transcription factors TEAD1-4 (Sd) [1,6,9]. These genetic studies have shown that in *Drosophila* the pathway exists in two activation states. The “on” state where the homologues of the core kinases are active and LATS1/2 can phosphorylate YAP and TAZ and sequester them in the cytoplasm. Phosphorylation of YAP on the Ser127 (TAZ Ser89) residue prevents YAP/TAZ-TEAD-dependent transcriptional activity ultimately preventing proliferation [1]. On the other hand, in the “off” state the MST1/2 and LATS1/2 are not active, and unphosphorylated YAP and TAZ translocate to the nucleus, bind to TEAD allowing the transcription of proliferation and pro-survival signals. While there is a wealth of evidence indicating the existence of a conserved Hippo pathway, delineation of the mammalian pathway demonstrated that there has been divergent evolution, as exemplified by the existence of several mammalian homologue isoforms of the *Drosophila* proteins [1,2]. Importantly, even before the description of the pathway in flies, several members of the pathway were shown to have independent functions, whereas other proteins have opposing functions as illustrated by the evidence that dRASSF is a Hippo inhibitor while its human homologues RASSF1A and RASSF2 are MST1/2 activators [10,11,12,13]. For the purpose of this review and to facilitate the reading we use the terms canonical Hippo pathway, which is commonly used in the scientific community, to refer to the mainstream view of the pathway that mainly include the homologues of the *Drosophila* pathway. The terms non-canonical MST/Hippo pathway/network is used for any other regulator and effectors of the pathway described so far that are not considered part of the canonical view.

Although the model of a linear, canonical signalling pathway is attractive, it is evident that this pathway is embedded in a complex signalling network. Numerous crosstalks with signalling pathways involved in cell fate regulation and cellular homeostasis such as the Ras/ERK, AKT, WNT, NOTCH, TGFβ, SHH and JNK pathways have been shown [10,14,15,16]. As recently discussed in an editorial in Science Signalling, the separation of signalling pathways into canonical/non-canonical arms can result in the oversight or even arbitrary dismissal of important findings that complete the understanding of biological processes [17]. In the case of the MST/Hippo pathway, the bulk of studies within the cancer setting have assigned YAP1/2 and TAZ to behave as oncogenes [1]. The interest in these putative oncogenes has already led to the screening for anticancer drugs that may target these proteins [18,19]. This idea ignores the evidence that YAP has also been described to have tumour suppressor properties by activating apoptosis [2,3,20,21,22,23,24]. As we approach the anniversary of the first decade of the mammalian Hippo pathway, we want to take the opportunity to review the knowledge that we have gathered so far in order to get a fuller understanding of all the functions of the pathway [25].

Apoptosis is the best characterised form of programmed cell death and is essential in the regulation of cellular homeostasis [26,27]. Although the signal transduction networks that regulate these physiological functions are complex, two main pathways have been defined depending on the origin of the activation signal [27]. The extrinsic pathway is activated by extracellular signals that activate the death receptor family of proteins which includes FAS (Fas cell surface death receptor), TNF (tumour necrosis factor) and TRAIL (tumour necrosis factor ligand superfamily) receptors resulting in the activation of the caspase cascade [27]. The intrinsic pathway is activated by DNA damaging agents such as ionizing radiation or chemotherapy agents that induce mutations in the DNA [28]. When the DNA repair machinery cannot correct the mutations there is an activation of the intrinsic pathway, which involves the depolarization of the mitochondrial membrane potential. This is followed by the release of several proteins that ultimately induce the activation of caspases [27] and members of the p53 family. Both pathways are closely related and share many common effectors. Core proteins of the MST/Hippo pathway appear to be part of the machinery that allows for the activation of apoptotic pathways [29]. Moreover, mice genetic models have confirmed *in vivo* that YAP mediates a pro-apoptotic signal in hepatocyte unless there is a secondary growth promoting signal [30]. Cell death triggered by pathway members cannot be explained by the canonical Hippo pathway. In particular, the role of a non-canonical MST/Hippo pathway in the activation of the classical apoptotic networks was illustrated early on by demonstrating that death receptors and DNA damaging agents activate core kinases of the pathway [13,31]. This non-canonical MST/Hippo pathway regulates p53 and p73 dependent cell death [3,24,32,33,34,35]. Importantly, rescue of YAP expression levels in haematological malignancies triggers p73-dependent apoptosis supporting the relevance of this non-canonical MST/Hippo pathway in human cancers [36]. In this review, we predominantly focus on how the non-canonical mammalian MST/Hippo regulates cell death. In addition, we briefly review evidence showing the regulation of cell death by the canonical pathway in *Drosophila* and non-canonical functions of the proteins of the pathway in the fruit fly. Finally we discuss the relevance of the pro-apoptotic signals mediated by the pathway in several pathological processes.

## 2. The Canonical *Drosophila* Hippo Pathway

**Figure 1:**
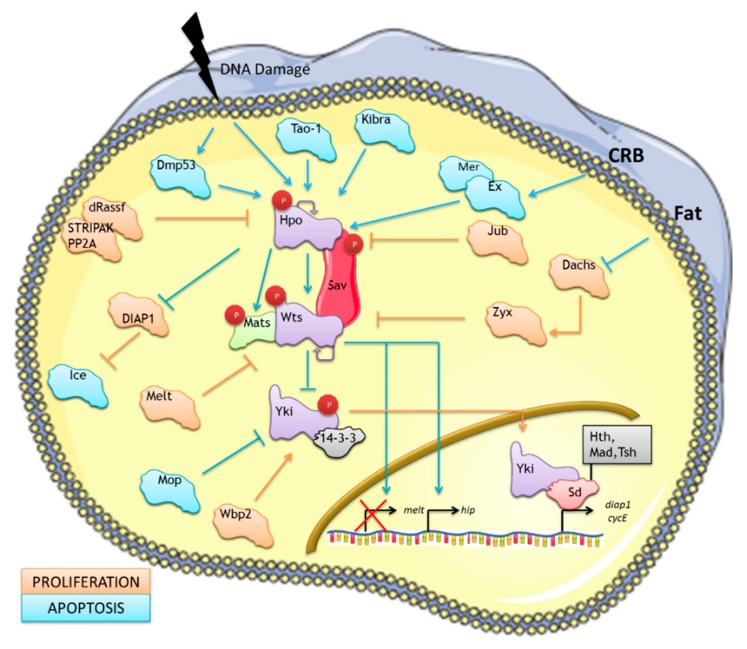
The regulation of Hippo signalling in *Drosophila melanogaster.* The scheme summarises the canonical Hippo pathway and some of the non-canonical functions described to date. The proliferative signals are represented in orange and the pro-apoptotic signals are represented in blue. The black box illustrates the newly described transcription factors binding to Yki.

The Hippo pathway is essential in cell proliferation control and regulation of apoptosis in *D. melanogaster* [4,37] by inhibiting a transcriptional co-activator protein, Yorkie (Yki) [38]. The coordination of those cellular processes must be precisely regulated in order to form organs with the correct number of cells and to avoid unrestricted growth [39,40]. Most of the components of the pathway are ubiquitously expressed, but in order to trigger cell death or cell cycle progression, the pathway activity is tightly regulated by complex molecular machinery [41]. The *Drosophila* Hippo pathway is extensively reviewed elsewhere [1,42] and Figure 1 summarises the core of the pathway and other proteins that are part of this network mentioned in this review. The regulation of the kinase cassette of the pathway is relatively well understood in *Drosophila*. Hpo and Warts are both Serine/Threonine kinases and are activated in response to different extracellular signals that increase Hpo kinase activity. Hpo in turn directly phosphorylates Wts. The interaction and activation of both kinases requires the participation of the scaffold protein Sav and the adaptor protein Mats. Active Wts phosphorylates its downstream effector Yki promoting the binding of this protein with 14-3-3 resulting in the sequestration of Yki to the cytoplasm [38]. When the kinases of the pathway are inactive, unphosphorylated Yki translocates to the nucleus where it binds the transcription factor Scalloped (Sd) [4]. The binding of Yki to Sd results in the transcription of pro-proliferative and anti-apoptotic genes such as *CYCE* and *DIAP* (Drosophila inhibitor of apoptosis) [38]. Thus, nuclear translocation of Yki is anti-apoptotic in this context since the target genes inhibit the activation of caspase-dependent cell death. Experiments using the *Drosophila* eye model, indicated that Hpo, Wts and Sav knockout results in unrestricted growth while overexpression of activated Yki resulted in hyperplasia. This led to the classification of Hpo, Wts and Sav as putative tumour suppressors and *Yki* as a putative oncogene [1].

Other proteins are now considered to be part of the pathway and each new discovery points to the existence of a complex signalling network rather than a linear pathway in *Drosophila* [37]. In particular, after the discovery of the core cassette of the pathway several other tumour suppressors were identified as regulators of the pathway using gene-based approaches, targeting Hpo and/or Wts [1,37]. These include the FERM domain proteins Merlin (Mer) and Expanded (Ex), the proto-cadherins Fat (Ft) and, the CK1 family kinase Disc overgrown (Dco), the WW and C2 domain-containing protein Kibra, the apical trans-membrane protein Crumbs (Crb), and Tao-1 [1,37,43]. Mutations of these upstream activators of the pathway lead to overgrowth phenotypes. As well as activators, upstream repressors were also identified. For example, dRASSF (Ras-association domain family) is able to antagonise the Hippo pathway by competing with the scaffolding protein Sav for its binding with Hpo through its SARAH domain [44], and recruiting a Hpo-inactivating PP2A complex called dSTRIPAK [45]. Conversely, several members of the mammalian RASSF proteins have been shown to activate the pathway, suggesting crucial divergences between the species [46]. Additional repressors of the pathway include the LIM domain-containing proteins Ajuba (Jub) [47], Dachsous (Dachs) and Zyxin (Zyx) [48]. These proteins are respectively able to facilitate cell proliferation by influencing the expression of CycE and DIAP1 by inhibition of Wts phosphorylation of Yki. Zyx is a downstream effector of the Fat branch of activation of the Hpo pathway and mediates Ft’s effect on Wts protein levels. Dachs (unconventional myosin) was shown to be another crucial protein which binds to Zyx facilitating its binding and inhibition of Warts, leading to activation of proliferative and anti-apoptotic genes. Dachs is recruited to the membrane by Fat where it can overlap with Zyx, allowing Fat to modulate Zyx-Warts binding, and inhibiting Zyx negative regulation of the Hpo-Warts kinase cascade. Importantly, this data shows that even though both Jub and Zyx, have a C-term LIM domain, they regulate the Hippo signalling in a very distinct manner [48]. Additionally some direct regulators of Yki have been identified. For instance Mop is another putative tumour suppressor that binds and inhibits Yki transcriptional activity [49] and Wbp-2 contributes to Yki-dependent transcription [50].

### 2.1. The Hippo Pathway Regulation of Pro-apoptotic Signals

The first articles that described the role of the proteins indicated that the involvement of the Hippo proteins in the activation of apoptosis might be more intricate. In particular, active Hpo was shown to promote the transcription of the pro-apoptotic gene *Head involution defective* (*Hip*) resulting in the activation of apoptosis [51]. Intriguingly, Harvey *et al*. showed that Hpo could phosphorylate DIAP *in vitro* and proposed it regulates DIAP protein levels by direct phosphorylation which induces ubiquitination dependent degradation [52]. Contemporary work from Pantalacci *et al.* also came to similar conclusions [53]. The work from this group showed that Hpo can also regulate cell death by direct phosphorylation of DIAP1 which would promote apoptosis by decreasing the protein level of DIAP1. Importantly, this work strongly indicated that this function of Hpo was independent of Wts. Finally, the authors also showed that in order to facilitate the association between Hpo and its binding partner Wts, Hpo is able to promote the phosphorylation and stabilisation of the tumour suppressor scaffold protein Sav [53]. Therefore, this early work already indicated that different feedback loops regulate the pathway and determine cell fate in the fruit fly. Importantly, work from Tapon’s group showed that the Hippo pathway also participates in stress-induced responses. Hpo can be activated by ionizing radiations (IR) in a Dmp53-dependent manner. Further, Hpo is required for the cell death response elicited by IR or the ectopic expression of Dmp53 [54]. Finally, the Hippo pathway has been shown to regulate activation of apoptosis via Yki and Dmp53 which control the expression of the pro-apoptotic gene *reaper* [55] These works show a direct relation of proteins of the *Drosophila* pathway with Dmp53 one of the most important regulators of cell death that remarkably is also a downstream effector of LATS1/2 [28].

### 2.2. Non-canonical Regulation of the Hippo Pathway: Evidence for the Existence of a Hippo Signalling Network

As indicated above, the canonical Hippo pathway belongs to a signalling network and numerous proteins have been shown to play a role in its regulation. Thus, the mapping of the *Drosophila* Hippo signalling network is still a work in progress. This was clearly illustrated by the results of a proteomics study in *Drosophila* which led to the identification of 153 proteins and 204 interactors within the Hpo signal transduction network [56]. This work clearly indicated that the proteins of the pathway are part of a wider signalling network that might mediate non-canonical functions of the Hippo pathway.

Importantly, further support for non-canonical Hippo pathway functions in *Drosophila* can be found in the literature [37]. For instance, the activation of the Hippo pathway is necessary for Wts-mediated functions in post-mitotic neurons [57]. In the fruit fly the R8 photo-receptor cells can either be specified to receive a long wavelength or a short wavelength. It has been shown that Wts and Melted (Melt) play opposing roles in defining the fate of the photoreceptor by directly controlling the regulation of the transcriptional activity of Melt [57,58]. In addition, the Hippo signalling core proteins have also been described to be involved in dendrite morphogenesis. Hpo is essential for the tilling and maintenance of dendrites whereas Wts and Sav are only required for dendrites maintenance, suggesting that each component of the pathway not only have functions independently of Yki, but, crucially, also from each other [59,60].

The existence of non-canonical functions can also be extended to Yki which may switch between different effectors to regulate specific functions. For example, there is evidence that Yki can also interact with and regulate other transcription factors such as Homothorax (Hth), Protein mothers against DPP (Mad) and Teashirt (Tsh) [47,61]. These data challenges the view that Sd is the sole transcription factor mediating Yki-dependent transcription. Nevertheless, the interaction of Yki with these effectors is cell and tissue type specific and is coordinated with Yki-Sd transcriptional activity [62]. Further study of these Yki effectors may result in a better understanding of the biological functions mediated by Yki. Intriguingly, it has also been described that Yki as well as DIAP1 have a non-canonical role in the regulation of the fruit fly epithelial tube size. Unexpectedly, Yki activity in developing embryos increases rather than decreases the length of the tracheal tubes. Yki controls cell shape during tracheal morphogenesis through the regulation of DIAP and Ice, a homologue of mammalian caspases, defining here a new non-apoptotic role of Yki [63]. Importantly, the regulation of Yki activity by Wts phosphorylation may be more complex than what the current model proposes [47]. The widely accepted view is that activation of the Hippo pathway concludes in the phosphorylation of Yki on Ser168 by Wts, which induces 14-3-3 binding and cytoplasmic retention [4]. Interestingly, work from Irvine’s group using live-cell imaging showed early on, that Yki regulation requires the phosphorylation of several residues aside from S168 [64]. These experiments showed that the YkiS168A mutant was not restricted to the nucleus and was still responding to Wts activity which pointed to the existence of additional phosphorylation residues regulated by Wts [65]. This data also indicated that Yki localisation and Yki-dependent transcription could be mediated by other kinases. Importantly, the existence of different phosphorylation statuses of Yki can explain how the binding to different transcription factors is regulated. Given the high level of conservation of elements of this network, future studies in *Drosophila* are likely to contribute towards our understanding of the mammalian network and shed light how this signal transduction network is involved in different pathologies.

## 3. Mammalian MST/Hippo Pathway, a Regulator of Cell Death at Many Levels

### 3.1. The Canonical Mammalian Hippo Pathway

In 2007 two papers showed the existence of a signalling pathway that included the human homologues of the Drosophila pathway MST2, LATS1 and YAP [3,4]. These articles were preceded by a series of publications that characterised some of the interactions of what has become the mammalian Hippo pathway [10,66]. These previous works together with the evidence from *Drosophila* were key in the fast delineation of the mammalian pathway and will be further explained in this review. Pan’s group, using mouse genetics, demonstrated a mammalian Hippo pathway closely resembling the *Drosophila* Hippo pathway. Thus, MST1/2 and LATS1/2 would be tumour suppressors that negatively regulate by direct phosphorylation YAP1, which would behave as an oncogene. The role of YAP and MST1/2, LATS1/2 and SAV1 (WW45) in the regulation of cellular homeostasis and organ size in several tissues was supported by the work from several groups published shortly after [67,68,69]. Thus, the use of genetic models demonstrated that the core of the Hippo pathway is conserved in mammals [67,68,69]. The interaction between YAP and TEAD, the mammalian homologue of *Sd*, had been already described [70], and soon after it was shown that the TEAD-YAP complex mediates MST1/2 and LATS1/2 dependent transcription [71]. These works were the basis for the canonical mammalian Hippo pathway. Increasing evidence shows that this pathway is more complex than originally described, indicating the existence of non-canonical regulation of the core proteins of the pathway [15,23,72,73,74,75,76,77]. The canonical Hippo pathway has been reviewed elsewhere, including this special issue [1,9] and we are only going to refer to it when there is direct relevance to the role of the pathway in cell death regulation.

### 3.2. The Non-canonical MST/Hippo Pro-apoptotic Pathway

**Figure 2:**
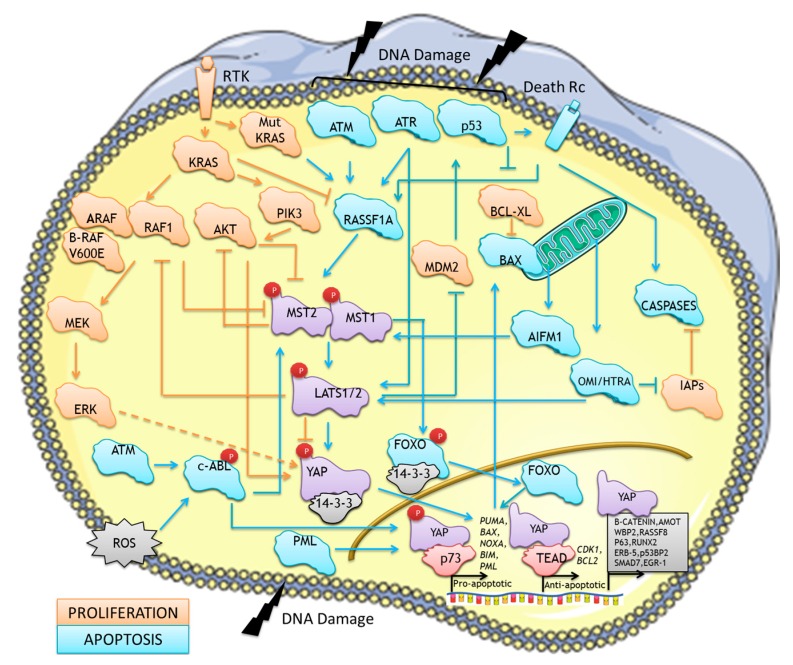
The cross-talk of mammalian Hippo signalling with the MAPK, PI3K and intrinsic and extrinsic apoptotic pathways. The scheme represents the MST/Hippo signalling network that regulates the pro-apoptotic signals mediated by the core proteins of the pathway. The proliferative signals are represented in orange, meanwhile the pro-apoptotic signals are represented in blue. The black box illustrates the described interactors binding to YAP.

Work published by our group, followed by works from two other groups [78,79], also demonstrated the conservation of the pathway through evolution but showed a divergent picture of the pathway as a direct mediator of pro-apoptotic signals [3]. The mapping of the pathway and characterisation of the molecular mechanisms by our group was done using sequential interaction proteomics [80]. This started with the identification, by O’Neill *et al.* of the proto-oncogene RAF1, a member of the MAPK pathway, as a direct interactor of MST2 [81]. This work showed that RAF1 binds to and inhibits MST2 in a kinase-independent fashion, therefore exhibiting a protective effect against apoptosis [81]. Previous work had shown that MST1/2 are activated by dimerization and auto-phosphorylation [11,82,83]. This work showed that RAF1 inhibitory binding prevents MST2 autophosphorylation and recruits a phosphatase that inactivates this kinase [81]. Our follow up studies, using mass-spectrometry based proteomics [80], identified the human tumour suppressors LATS1 and RASSF1A as MST2 interactors that mediated the pro-apoptotic signal downstream of RAF1 upon apoptotic stimuli [3]. The interaction between RASSF1A and MST2 had already been described by Avruch’s group [83,84] which indicated that RASSF1A prevented MST2 activation. Conversely, other groups had showed that RASSF1A is an activator of MST2 kinase activity [85,86]. Our own work indicated that RASSF1A dissociates the RAF1-MST2 complex which results in the activation of MST2 kinase activity [3]. We also showed that RASSF1A binds MST2 in the SARAH domain, which overlaps with the RAF1 binding region indicating that RASSF1A and RAF1 have mutually exclusive association to MST2. Further to this, results from this study showed that RAF1 has the ability to counteract RASSF1A-induced apoptosis and that this effect was independent of its ability to activate the MEK-ERK signalling module [3]. Upon re-expression of RASSF1A in MCF7 cells or activation of death receptor by FAS ligand in HeLa and MCF7 cells we demonstrated that MST2 bound to RASSF1A interacts with endogenous LATS1 [3]. RASSF1A scaffolds the interaction between both proteins and this complex activates cell death [3]. Led by previous studies performed in *Drosophila* we investigated if LATS1 would interact with YAP1. We identified an association between YAP1 and LATS1 and could show that this interaction is regulated by RASSF1A. It was confirmed that this LATS1-YAP1 association was MST2 dependent, illustrating that YAP1 was a component of the RASSF1A-MST2 pathway [3]. However, contrary to what was known at the moment in *Drosophila* our work clearly showed that LATS1 phosphorylation of YAP1 resulted in a decrease of LATS1-YAP1 interaction and promoted translocation of YAP1 to the nucleus. We did not show that LATS1 phosphorylated YAP1 on Ser127. In fact, our kinase assays indicated that different YAP1 residues were phosphorylated by LATS1 upon RASSF1A-MST2 activation which would be similar to observations in *Drosophila* and other mammalian systems [3,65]. The completion of the delineation of the pathway was facilitated by the seminal works from the groups of Strano and Blandino [24] and Basu and Downward [87] that had identified YAP1 as a co-transcriptional regulator of p73, a member of the p53 family of tumour suppressors. Based on these works we showed that upon translocation to the nucleus YAP1 binds p73 and causes the transcription of the pro-apoptotic gene *PUMA* [3], as illustrated in Figure 2. Another important finding of this work was that RASSF1A-MST2-LATS1 complex mediated FAS dependent apoptosis in a YAP-p73 independent fashion. This clearly confirmed previous observations that FAS regulates the RASSF1A-MST1/2 interaction [85], and indicated the existence of different pro-apoptotic effectors downstream of LATS1. FAS activation of the pathway also indicated that the novel pathway that we had described, crosstalks with the classical extrinsic apoptotic pathway. Importantly, our work clearly indicated that, as previously pointed out by the work of Strano [23,24,66,88], in human breast cancer cells YAP1 could be a tumour suppressor. This was confirmed by the analysis of clinical data from breast cancer patients that indicated that patients that express higher level of *YAP* mRNA have better survival rates that those patients that lose expression of this mRNA [3]. This observation was later confirmed by other clinical studies that monitored YAP protein expression, which is in strong support for the tumour suppressor role of YAP in humans [20,89]. Shortly after the publication of this study, independent work from Kawahara came to a similar conclusion as our work, demonstrating that LATS2 also regulates YAP-p73 pro-apoptotic signalling and that this requires the phosphorylation of the Ser127 residue [78]. This work also supported the possible relevance of this pathway in cancer since the authors showed that it can mediate chemosensitivity of leukemic cells [78]. This work further linked the pathway to the other classical apoptotic pathway, the intrinsic pathway and DNA damage response. Finally, a third paper by Sudol’s group further confirmed the relationship between the core of the MST/Hippo pathway and p73 but paradoxically, in contrast with the observation of Kawahara, the authors showed that LAST1 phosphorylation of YAPS127 inhibited the interaction between YAP and p73 and prevented pinocytosis [79]. Despite this difference that might indicate different regulatory mechanisms, this paper also confirmed the existence of the novel, pro-apoptotic MST/Hippo pathway. In summary these early works showed the existence of a pro-apoptotic signal mediated by the RASSF1A-MST2-LATS1/2-YAP1/2-p73 pathway which might be relevant for different human tumours.

### 3.3. From Pathway to Network

Since the description of the MST/Hippo pathway, several groups have extended the evidence for the involvement of these proteins in the regulation of apoptosis. As it has happened with other signalling pathways the picture that is emerging shows that the regulation of cell death by the pathway is mediated through a complex signalling networks [90]. We still lack a complete understanding but it appears as if these signalling networks include some of the main regulators of cell homeostasis such as p53, RAS, AKT, WNT and caspases [32,33,34,91,92,93]. Several of the proteins in the core MST/Hippo pathway act as signalling nodes that regulate cell death through different mechanisms allowing a tight regulation of pro- and anti-apoptotic signals [11,13,23]. Importantly, the activating stimuli of the pro-apoptotic MST/Hippo pathway are better characterised than the activators of the canonical pathway and include both the death receptors and the DNA damage response [66]. This clearly indicates that the pathway is closely integrated with the extrinsic (activated by death receptors) and the intrinsic (activated by DNA damage) [26] apoptotic pathways. Thus, in order to understand the role of the MST/Hippo pathway in the regulation of cell death we need to understand the wider signalling networks. In this part of the review we try to summarise key findings of what we know to date of the pro-apoptotic MST/Hippo signalling network focussing on interactors that regulate the pathway upon pro-apoptotic signals.

#### 3.3.1. Regulators

##### RASSF Family

The RASSF family is formed by 10 genes (*RASSF1–10*) with some giving rise to multiple isoforms via alternative splicing and distinct promoter usage [94]. These proteins lack enzymatic activity and seem to be classical scaffold proteins. The main structural feature of the proteins of the family is their RA (Ras-association) domain which potentially allows them to interact with the RAS family, however many of the 10 members have yet to be shown to interact with RAS GTPases [95]. RASSF1–6 form the C-terminal RASSF subfamily which are characterised by the presence of the SARAH (SAV/RASSF/Hpo) domain, located at the C-terminus of these proteins [28]. This domain is involved in protein-protein interactions with MST1/2 [96,97]. Several lines of evidence show that RASSF1C, RASSF2, NORE1A (RASSF5), RASSF4 and RASSF6 regulate the pro-apoptotic signal mediated by MST1/2 [28]. In fact, proteomics experiments performed by different groups showed the interaction of MST1/2 with RASSF1-5 [72,98,99,100]. The role of the different members of the C-terminal RASSF family in the regulation of MST1/2 and the MST/Hippo pathway is still poorly understood but it is likely that these proteins are key regulators of the apoptotic signal mediated by the pathway. Importantly, while most of the members of this family seem to be activators of MST1/2 pro-apoptotic activity, other members of the family such as RASSF1C and RASSF6 have been shown to inhibit MST1/2 pro-apoptotic signal. As described above, the role of the scaffold RASSF1A tumour suppressor as a regulator of the MST/Hippo pathway is the best understood [3]. RASSF1A is a splicing isoform of *RASSF1* and is one of the most commonly deregulated genes in cancer [46,101,102]. This protein regulates the activation of apoptosis mediated by death receptors and DNA damaging agents. As mentioned above, this contrasts with the observation that in *D. melanogaster* dRASSF, which is the only homologue of the C-terminal RASSF family of proteins identified, antagonises the Hippo pathway. This evolutionary divergence has been used for excluding RASSF proteins as canonical regulators of the mammalian pathway or cast doubts about the physiological relevance of the regulation of MST1/2 by the pathway [1]. The role of the RASSF family members as activators of MST1/2 is supported by strong evidence from several groups [2,28], however it must be noted that the first experiments performed by Avruch’s group showed that RASSF1A and RASSF1C inhibited MST1 kinase activity which could be an indication of a dual function of RASSF proteins in the regulation of MST1/2 proteins [83]. This would be supported by evidence that show that RASSF1C prevents the activation of MST1/2 dependent apoptosis and behave in some tumours as an oncogene even if it can still activate MST2 kinase activity [103,104]. Another possibility that could explain these early results is that these proteins are scaffolds and over-expression of this class of proteins beyond optimal conditions can have the opposite effect on the function of their interactors [105]. If the original experiments were performed with extremely high concentration of RASSF1A plasmids than in subsequent experiments this would have resulted in the inhibition of MST2 activity. RASSF2 is another tumour suppressor silenced in renal cell carcinoma that was shown to bind and regulate the activation of MST1/2 and initiation of apoptosis, although it is not known whether this apoptosis is mediated by the MST/Hippo pathway [100]. The other member of the family that has been shown to be an activator of MST1 is NORE1A [106,107]. These works showed that this scaffold regulates the activation of MST1/2 pro-apoptotic signal downstream of Ras. Interestingly, it has been shown that NORE1A regulates the activation of the pro-apoptotic MST1/LATS1 signal in osteosarcomas cells through the activation of p53 [108]. Intriguingly, the regulation of the proteins of the pathway might not be always mediated by their interaction with MST1/2. A recent report has shown that RASSF1A might directly regulate YAP1 through RhoB, although no experiment was done to see whether this requires MST1/2 and LATS1/2 or it would be two convergent mechanisms that regulate this co-transcription factor [109].

The implication of the *RASSF* family of scaffolds in the MST1/2 Hippo pathway seems to extend to the other members of the N-terminal subfamily. The members of this subfamily do not contain a SARAH domain and have not been shown to bind to MST1/2. However, RASSF7 and RASSF8 have been shown to interact with YAP1 in proteomics studies [98,110] and in the case of RASSF8 this interaction has been experimentally validated [110]. However further studies to decipher the roles of these proteins in the regulation of the MST/Hippo pro-apoptotic pathway are warranted.

##### Family of RAS GTPases

The original work that described that RASSF1 and NORE1 as regulators of MST1 showed that the formation of the RASSF1-MST1 and NORE1-MST1 complexes was regulated by the members of the RAS family [83,111,112]. In the same work the authors identified NORE1A but not RASSF1 as an interactor of RAS proteins with higher affinity for KRAS [83]. Importantly, it was shown that the NORE1-MST2 complex activated a pro-apoptotic signalling pathway downstream of KRAS. Although the authors failed to show a direct interaction between RASSF1 and RAS proteins, they also showed that the complex RASSF1A-MST1 is regulated by this small GTPase. The interaction with KRAS would require the participation of NORE1 [112]. In light of this data, after we described that RASSF1A is a direct activator of the MST/Hippo pathway, we moved forward to study if this pathway was the elusive effector pathway downstream of KRAS that transmits the pro-apoptotic signalling mediated by this oncogene [113]. Our work showed that KRAS is a regulator of the MST/Hippo pathway and helped to shed light on how RASSF1A regulates the pathway [114]. Importantly, unlike the previous works from Avruch’s group, we and Donninger saw that KRAS proteins bind to RASSF1A [46,114,115]. In particular, we demonstrated that the binding is specific for the KRAS isoform in HCT116 colorectal cells that do not express NORE1 [114]. Interestingly, no endogenous interaction between RASSF1A and either HRAS or NRAS was observed, which suggests that KRAS is the only RAS isoform that can bind to RASSF1A [115]. Findings from this study demonstrated that KRAS selectively interacts with RASSF1A in a receptor tyrosine kinase dependent fashion, and MST2 could also be detected in this complex [115]. Interestingly, we showed that KRAS has a paradoxical effect on the activation of the pro-apoptotic pathway [114,115]. EGF activation of wild type KRAS promotes the interaction of RASSF1A and MST2 but does not result in an activation of LATS1. On the other hand, sustained KRAS signal as that triggered by mutant KRAS promoted the binding of MST2 and LATS1 and the activation of apoptosis independent of YAP. This data confirmed that in mammalian systems there are several effectors that mediate LATS1 dependent apoptosis. Using proteomics we identified Mdm2 as a LATS1 interactor in cells that expressed mutant KRAS. This finding immediately caught our attention since work from Aylon *et al.* had shown that p53 is regulated by LATS2 in an oncogenic HRAS dependent fashion [33]. Similarly, we saw that LATS1 regulates p53 transcriptional activity. Our data showed that mutant KRAS dependent activation of the MST2-LATS1 kinase cassette results in the sequestration of Mdm2 by LATS1 and subsequent stabilization and activation of the p53 tumour suppressor. Interestingly, we saw that the wild-type *KRAS* allele counteracts the pro-apoptotic effects of mutant *KRAS* and prevents the oncogenic-dependent apoptosis. Altogether this study showed that the anti-apoptotic effect of the wild-type *KRAS* allele worked in parallel with EGF-R signaling [32]. Interestingly, mutant KRAS initiates an autocrine feedback loop that activates the EGF-R and wild-type *KRAS* allele to inhibit the MST/Hippo and p53 dependent apoptosis. Moreover, we also showed the possible clinical relevance of these findings in 2 cohorts of colorectal cancer patients. Thus, we showed that the MST/Hippo pathway is a part of a signalling network highly deregulated in human cancer that includes KRAS and p53 [114,115]. The relation of the RAS proteins with the MST/Hippo pathway and the physiological relevance of this was subsequently supported by the work from several groups that showed that Ras proteins can regulate YAP proliferative signal (*i.e.*, canonical activity) by repressing the core kinases of the pathway [116]. Several works have shown that YAP can mediate oncogenic KRAS in different human tumour types [117,118] and even bypass oncogenic KRAS oncogenic addiction in pancreatic cancer [119]. This relation between KRAS and YAP may have an implication in the development of novel therapies against *RAS* mutant tumours [120]. Thus, the MST/Hippo pathway seems to have several roles in KRAS-dependent tumours.

##### RAS Effector Signalling Network

Further evidence from different groups supports the idea that this pathway is at the cross-road of proteins involved in cellular homeostasis. In particular, different lines of evidence indicate that this pathway is closely related to two of the best characterised RAS effectors, the MAPK (ERK) pathway and the PI3K/AKT pathway [14]. Since RASSF1A is also an effector of RAS, these three RAS effector pathways form a network that is tightly regulated to secure appropriated cell fate decision. The cross regulation of the pathways appears bidirectional (Figure 2) and several lines of evidence suggest that the loss of this cross regulation is necessary for RAS dependent cellular transformation [14].

The crosstalk of MST/Hippo proteins with the MAPK pathway was evident from our early works since we had already shown that RAF1 is a direct regulator of the pathway [81,121]. Additionally, it was shown that ARAF but not BRAF also binds to MST2 and prevents its activation [122]. Both members of the RAF family bind to MST2 through the amino acids 151 and 303 of RAF1 [15], a region that diverges among the RAF isoforms, explaining the lack of interaction with BRAF. Surprisingly, although no interaction was observed between MST2 and BRAF [81] it was shown that BRAFV600E, the most common oncogenic mutant of BRAF, binds to the C-terminal of MST1 and inhibits its kinase function in papillary thyroid carcinoma [123]. This suggests that, at least in this cancer type, inhibition of the pro-apoptotic MST/Hippo pathway is necessary for BRAFV600E dependent transformation and supports the idea that both pathways are part of the same signalling network. Importantly, the crosstalk between the MST/Hippo pathway and the MAPK pathway is mediated at different levels since the formation of the MST2-RAF1 complex prevents the binding of RAF1 to MEK and LATS1 can inhibit RAF1 kinase activity by a feedback loop [15]. When RAF1 is phosphorylated on Ser259 it cannot activate the ERK pathway. This site must be dephosphorylated before RAF1 can be phosphorylated on Ser338 as part of the normal physiological activation of the kinase [124]. However, Ser259 phosphorylation promotes RAF1-MST2 complex formation, indicating that dephosphorylation of Ser259 promotes both proliferative and apoptotic pathway activation. This study showed that LATS1 is a Ser259 kinase; therefore, LATS1 kinase is involved in regulating both the MST2 and ERK pathways [15]. This further illustrates how the MST2 pathway is closely integrated with the MAPK pathway at numerous levels. Further illustration of this comes from the observation that ERK affects the YAP-TEAD complex by regulating YAP phosphorylation downstream of oncogenic KRAS in pancreatic ductal carcinoma [125]. Thus, YAP itself seems to be an effector of oncogenic KRAS [125].

The cross regulation of the MST/Hippo pathway with the RAS effector PI3K/AKT pathway is mediated by different mechanisms [14]. AKT is the main downstream effector of phosphoinositide 3-kinase (PI3K), and this proliferative pathway is deregulated in many cancers [126]. Two independent studies showed that MST1/2 pro-apoptotic signalling is negatively regulated due to direct phosphorylation of MST2 by AKT [93,127]. MST2 is phosphorylated by AKT on at least two residues (Thr117 and Thr384) which inhibit the pro-apoptotic function of MST2. The two phosphorylation sites occur at both the C- and N-terminus of MST2 and therefore independent of its binding to RAF1. It was illustrated that this AKT-induced phosphorylation of MST2 inhibited its function via three main mechanisms: (i) promoting its interaction with RAF1 resulting in formation of the inhibitory complex, (ii) blocking the recruitment of MST2 by RASSF1A; and (iii) causing a direct inhibition of MST2 kinase activity [93]. Importantly, oncogenic KRAS also promotes the phosphorylation of MST2 by AKT which results in an inhibition of the pro-apoptotic signal mediated by the MST/Hippo pathway [32]. Additionally, MST1 can regulate the PI3K/AKT signalling pathway through the inhibition of AKT by direct phosphorylation [128]. Phosphorylation of AKT by MST1 results in an inhibition of its kinase activity and prevention of the prosurvival signal mediated by this protein. Further cross-regulation between the pathways seems to occur at the level of YAP. Early work showed that AKT can phosphorylate YAP and inhibit the p73 pro-apoptotic signal. Interestingly, AKT phosphorylates YAP at Ser127, promoting its association with 14-3-3 and retention of YAP in the cytoplasm where it is therefore unable to act as a coactivator of p73, attenuating p73 induced cell death [87]. This regulation of the MST/Hippo pathway by AKT might be particularly relevant in cancers that exhibit loss of PTEN a frequently lost tumour suppressor that inhibits the PI3K/AKT signalling pathway [129,130]. These cells already express high basal levels of AKT kinase activity and therefore may in turn suppress the MST2 pathway. Interestingly, YAP has been shown to downregulate the expression of PTEN [29] which should result in the inhibition of MST1/2 pro-apoptotic signal due to AKT inhibition.

All the evidence gathered so far indicate that the Hippo pathway forms a signalling network with the MAPK and AKT pathways that has a central role in the regulation of cellular homeostasis.

##### ATM and ATR

These proteins are the initial kinases that trigger the intrinsic pathway in response to DNA damage. Both kinases seem to mediate the regulation of the RASSF1A-MST2-LATS1 module by ionizing radiation (for a complete review see [27,131]). The work from O’Neill’s group demonstrated that after DNA damage ATM phosphorylates RASSF1A on Ser131 which triggers recruitment and activation of MST2 and LATS1 [132]. Subsequently, activated LATS1 promotes the activation of the pro-apoptotic program mediated by the YAP-p73 complex [132]. This group also showed that activation of ATM and ATR caused by DNA fork stalling activates the RASSF1A-MST2-LATS1 pathway [133]. In this case the activation of the pathway promotes the binding of active LATS1 to CDK2 which negatively regulates the kinase activity of this protein preventing the phosphorylation of BRCA2 in order to prevent chromosome instability and ultimately resulting in the activation of cell death [133]. ATR can also regulate the pathway through its substrate CHK1. CHK1 is a kinase that phosphorylates both RASSF1A and LATS2. When LATS2 is activated by CHK1 it prevents the degradation of p53 by sequestering Mdm2 which triggers p53 dependent apoptosis [131]. Importantly, ATM also activates the non-receptor Tyr kinase c-ABL by direct phosphorylation of the Ser465 residue. C-ABL has been shown to be a key regulator of YAP-p73 dependent apoptosis [134] and it also regulates the pro-apoptotic signal mediated by other core proteins of the MST/Hippo pathway as will be discussed below. In summary, the data gathered so far indicates that ATM and ATR can play a role as upstream regulators of the non-canonical MST/Hippo pathway that mediate the activation of the pathway by DNA damage.

#### 3.3.2. Core Kinases, at the Crossroad of Many Pathways

MST1/2 and LATS1/2 are part of a kinase cassette conserved through evolution. It is worth noting that the other members of both families MST3/4 and NDR1/2 also form a kinase module conserved from *Drosophila* known as the NDR pathway. The NDR pathway has also been shown to regulate cell fate in coordination with the proteins of the Hippo pathway but due to space limitations we will not discuss this here, for a review see [135,136]. Although the functions of MST1/2 and LATS1/2 are closely related, they also have independent functions from each other and are signalling hubs that mediate different signal transduction pathways. In addition to cell death that is the focus of this review, these proteins have been shown to be involved in other biological functions such as migration, differentiation and cell cycle progression [11,13,137]. Importantly, MST1/2 and LATS1/2 have been shown to regulate cell death upon different stimuli and through several mechanisms showing the diversity of the signalling network formed by these kinases.

##### MST1/2 Apoptotic Network

The Ser/Thr kinases MST1 and MST2 are part of the Ste20 kinase family and were cloned in 1995 by Chernoff’s group [11,138,139]. Both proteins are closely related and show sequence homology of 77% [79]. The catalytic domain of these proteins is situated in the N-terminal region while the C-terminal region contains and inhibitory region and the protein-protein interaction SARAH domain [10,140]. The protein sequence also contains caspase target sequences and caspase cleavage produces a constitutively active 36 kDa N-terminal kinase [141]. The regulation of full length MST1 and MST2 kinase activity requires the phosphorylation of several residues by different kinases including auto-phosphorylation [142]. MST1 and MST2 homo- and heterodimerise and facilitate the auto-activation of their kinase activity [142]. Knock-out mice of *MST1* and *MST2* are viable while the double knock-out is embryonic lethal indicating that both proteins have a high degree of functional redundancy in development [11]. Despite this redundancy there is evidence that support isoform specific functions of MST1 and MST2. For example, it has been shown that MST2 specifically mediates p73 dependent apoptosis in photoreceptor cells during retinal detachment [143].

Since the cloning of *MST1* and *MST2* it was clear that these proteins have pro-apoptotic functions [11]. Before their participation in the MST/Hippo pathway was described, several groups identified different molecular mechanisms that mediate MST1 and MST2 dependent apoptosis. First it was shown that activation of both proteins induces apoptosis through a caspase dependent and independent mechanism that requires JNK signalling [144]. Upon stress and FAS ligand stimulation MST1 was shown to be cleaved by caspase 3 and the N-terminal kinase MST1 seems to behave as a MAPKKK that activates MEKK1 and MKK7 [141]. Later it was also shown that after caspase proteolysis the N-Terminal constitutively activated kinase translocates to the nucleus and triggers apoptosis by phosphorylating histone H2B [145]. The cross-regulation of MST1/2 and caspases was shown to be more complex since MST1/2 can also mediate FAS-dependent caspase activation and therefore triggers the activation of the pro-apoptotic signals [31,141]. Other death receptors have been shown to regulate MST2 kinase activity. For instance it was observed that the ligand mediated activation of TNF1 resulted in recruitment of RASSF1A and in increased MST1/2 kinase activity [146]. Thus the regulation of MST1 and MST2 kinases by the activation of the death receptor seems to be controlled by different mechanisms including promoting the formation of the RASSF1A-MST1/2 complex [2]. As mentioned above, we showed that FAS-L triggers the activation of the pathway by promoting the binding of RASSF1-MST2-LATS1 and promoting the activation of apoptosis in a caspase independent fashion [3,81]. Thus, it would be important to get a better understanding of how the signalling network regulated by MST1/2 (and RASSF1A) is activated by death receptors. Different lines of evidence suggest that RASSF1A function must be regulated by the FAS DISC, the protein complex that conveys the death receptor signals and death receptor domains in TNF and TRAIL receptors [147]. Both RASSF1 and MST1 were shown to bind DAXX and DAP4, two death domain containing proteins [148,149]. Intriguingly, RAF1 was also proposed to be regulated by RIP2, another component of the DISC, which could be related to the mechanism that regulates the rescue of MST2 from RAF1 inhibitory binding by RASSF1A [150]. The regulation of RASSF1A by death receptors is also mediated by its association with MOAP-1 a protein that binds the death domain of TNF-R1 and promotes RASSF1A apoptosis through the regulation of the pro-apoptotic BH3 protein BAX [133].

In addition to the regulation of caspases, MST1 and MST2 regulate apoptotic pathways by interacting with apoptotic proteins in response to different signals. For example, MST2 has been shown to interact with Apoptosis-inducing factor (AIF), which is involved in caspase-independent programmed cell death. DNA damage was shown to promote the interaction between these proteins enhancing MST2 phosphorylation at T180 in renal cell carcinoma [151]. Furthermore, diabetogenic signals induce the activation of MST1 by auto-phosphorylation [152]. Active MST1 has been shown to directly phosphorylate the pro-apoptotic protein BIM in pancreatic β-cells. Phosphorylation of BIM results in the initiation of the caspase cascade triggering a positive regulatory loop where active caspase-3 cleaved MST1 initiates other MST1 dependent mechanism of apoptosis [153]. These, and experiments in animal models, indicate that this role of MST1 is central in the death of β-cells that is characteristic of both types of diabetes [152]. Similarly, MST1 phosphorylation of BCL-xL inhibits its anti-apoptotic effect, which results in cardiac myocyte apoptosis [154]. Finally, MST1 has been shown to promote apoptosis though p53 upon DNA damage [155]. Treatment with the genotoxic agent etoposide activates MST1 kinase activity. Active MST1 then inhibits the enzymatic activity of the deacetylase SIRT1 by direct phosphorylation. As a result, there was a decrease in the interaction between p53 and SIRT1 and an increase of acetylated p53, therefore initiating the transcription of pro-apoptotic genes resulting in apoptosis [155].

The other MST1/2 dependent pro-apoptotic signalling pathway that has been extensively studied is the MST1-FOXO pathway [11]. The transcription factors FOXO1 and FOXO3 regulate the transcription of several pro-apoptotic proteins and were identified as MST1 substrates [156]. FOXO proteins are phosphorylated by MST1 which prevents the binding of 14-3-3 and allows the translocation of FOXO1/3 to the nucleus [157]. Upon translocation, FOXO1/3 activates a pro-apoptotic transcription program that includes *NOXA* and *BIM* [158,159]. Importantly the MST1 phosphorylation of FOXO3 is prevented by an inhibitory phosphorylation of MST1 by AKT1 in neurons [160], which shows again the mutual antagonistic regulation of these two proteins. The functional relevance of this signalling pathway seems to be especially important in neurons and its deregulation can be associated with neurodegenerative diseases. Thus, oxidative stress has been shown to regulate motor neuron death through the phosphorylation of FOXO1 by MST1 in amyotrophic lateral sclerosis (ALS) animal models [161]. Similarly, the activation of MST1 and MST2 in neurons has been shown to be mediated by direct phosphorylation of the Y433 residue by c-ABL that reduces the interaction between MST2 and RAF1 [162,163]. Importantly, the activation of c-ABL is triggered by oxidative stress which is a common feature of ALS. The MST-FOXO pathway seems to be also deregulated in Alzheimer’s disease [158]. β-Amyloid accumulation in neurons activates the pathway, which leads to the transcription of *BIM* and the activation of apoptosis. Importantly, the regulation of the MST-FOXO1 pathway is not only restricted to neuronal cells as it has also been seen that in Jurkat T lymphoma cells FOXO activates apoptosis downstream of MST1 by promoting the expression of the BH3 pro-apoptotic protein NOXA [159].

The role of MST1/2 is not limited to the regulation of apoptosis. Loss of *MST1* and *MST2* has been shown to prevent autophagy in different species. Hansen’s group showed that both kinases can regulate this cell fate by direct phosphorylation of LC3, a key protein in the formation of autophagosomes [164]. On the other hand, MST1 was shown to be involved in the activation of necrosis in myocytes in a cardiomyopathy model [165]. Understanding the signals that regulate the activation of all these different cell death programs by MST1/2 could have a big impact on the treatment of different pathologies.

##### LATS1/2 Pro-apoptotic Network

*LATS1* and *LATS2* are also emerging as important signalling hubs that are related to the classical extrinsic and intrinsic apoptotic pathways both downstream of MST1/2 and through MST/Hippo pathway independent mechanisms. These genes were identified by two independent groups as homologs of Wts [166,167]. LATS1 and LATS2 are Ser/Thr kinases and belong to the AGC family [13]. The C-terminal kinase domain of these proteins have sequence homology of 85% [168] indicating that they may share common substrates. However the overall sequence homology between the two proteins is only of 52% which may account for the existence of functional differences [13]. Both proteins possess PPxY motifs that mediate its binding with WW domains. The kinase activity of both proteins is activated by MST1/2 through direct phosphorylation [13]. In addition to cell death, these proteins have been shown to regulate genetic stability, cell cycle progression and transcription [149]. Although these proteins have redundant functions they also have isoform specific functions [13].

As mentioned above, the MST/Hippo pathway pro-apoptotic signal is not only mediated by YAP-p73 but it is also by p53 [23]. How LATS1/2 coordinate these different effectors is still not clear but it might depend on the pro-apoptotic insults that trigger the activation of the pathway [23]. The existence of other, yet unidentified, LATS1 effectors are clearly indicated by the fact that FAS activation of the pathway is not mediated by p53 or YAP [3]. Additionally, LATS1 and LATS2 have been shown to be also closely involved in the regulation of the classical apoptotic pathways. LATS1 is activated by death receptors downstream of RASSF1A and MST2 [3]. FAS regulates the expression level of LATS1 by preventing the binding of this kinase to the E3 ubiquitin ligase ITCH [167]. The involvement of these kinases in the intrinsic pathway is also demonstrated by several lines of evidence and both isoforms might have specific functions in the regulation of this pathway [13]. Thus, in addition to the stabilization of p53, LATS1 was shown to increase the expression of the pro-apoptotic protein BAX [169]. Another protein of the intrinsic pathway that has been shown to be regulated by LATS1 is Omi/HtrA2 [170]. Omi/HtrA2 is a mitochondrial protein that is released to the cytoplasm following mitochondria depolarization induced by DNA damaging agents [26]. The mechanism by which LATS1 regulates this protein is better characterised. Thus, it was shown that LATS1 was able to bind, via its C-term region, to the PDZ domain of Omi/HtrA2, enhancing the protease activity of Omi/HtrA2, which results in a reduction of the level of expression of the anti-apoptotic protein XIAP [170]. Downregulation of XIAP results in caspase activation and activation of apoptosis. LATS1 also activates Omi/HtrA2 caspase independent apoptosis by increasing its protease activity [170]. In the case of LATS2, this kinase was shown to downregulate the expression of the anti-apoptotic proteins BCL-xL and BCL2 by a mechanism that is yet to be described but that requires LATS2 kinase activity [171].

Finally, the relation of LATS1/2 with p53 seems to be central in the regulation of apoptosis by these kinases and is closely related to the involvement of both kinases in cell cycle progression [169,172]. LATS1 and LATS2 have been shown to be involved in the regulation of mitotic check points including the spindle mitotic check point and the G1-tetraploidy check point. These two check points are thought to be closely related and prevent chromosomal haploidy [173]. The involvement of LATS1/2 in this check points is mediated by p53. It is known that p53 arrests cell cycle progression if a chromosomal imbalance is detected due to defects in spindle assembly. Prolonged cell cycle arrest results in the formation of a tetraploid cell due to exit of the cell cycle without chromosome segregation and cytokinesis [33]. When the resulting tetraploid cell enters mitosis the G1-tetraploidy check point arrests mitosis and in normal conditions this cell undergoes p53-dependent apoptosis. The mechanism by which LATS1/2 induce apoptosis has been shown to involve the stabilisation of p53 expression by inhibition of Mdm2 [33]. Moreover, in tetraploid cells p53 can also promote the expression of LATS2 in a positive feedback loop [33]. Importantly, mitotic stress induced by oncogenic HRAS also has been shown to induce apoptosis through the LATS2-p53 complex. Expression of HRASV12 activates ATM-CHK2 which increases the level of expression of LATS2 and results in the activation of p53-dependent apoptosis [35].

#### 3.3.3. YAP the Most Famous Effector of the MST/ Hippo Pathway

The transcription regulator YAP is the most studied member of the MST/Hippo pathway. This gene was identified as a YES kinase interacting protein by Sudol in 1995 [174]. There are splicing isoforms that contain 1 or 2 WW protein-protein interaction domain. YAP is regulated by post-translational modifications and can bind to and regulate several transcription factors. There are five LATS1/2 phosphorylation motif in human YAP1 Ser61, Ser109, Ser127, Ser164 and Ser397 [175] (four in the case of TAZ [176]), and these kinases have been shown to differentially phosphorylate these residues depending on different signals [177]. Among the known interactors are p53BP2, SMAD7, ERB-5, RUNX2, TEAD1-4, PEBP2a, p63, RASSF8, p73, WBP2 and AMOT [1,2]. This number is rapidly increasing as a result of several proteomics screens [110]. Thus, YAP seems to be at the center of several signalling pathways, and yet, we are still far from understanding how YAP coordinates all these different signals.

As explained above, several groups showed that YAP activates apoptosis in mammalians systems through the regulation of p73 [14]. It must be noted that in addition to the mechanisms described so far YAP-p73-dependent apoptosis was also shown to be regulated by the tumour suppressor PML [88]. Thus, it was shown by Lapi *et al.* that upon treatment with DNA damage agents, PML and YAP directly interact which results in YAP stabilisation. The YAP-PML complex form a complex with p73 that promotes the transcription of several pro-apoptotic genes including BAX and PML itself [88]. Interestingly, YAP-PML complex was shown to be able to regulate p53-dependent senescence downstream of ATM [178]. Additionally YAP can contribute to the activation of apoptotic programs through the regulation of different transcriptions factors. For example, it was shown that YAP-1 binds to EGR-1 in prostate carcinoma cells upon irradiation of the cells [179]. The EGR-1-complex was shown to increase the transcription of the pro-apoptotic genes *BAX* and *PUMA*, which resulted in an increase of apoptosis. The binding of YAP1 with its different effectors is not well understood, but it is likely regulated by a combination of different post-translational modifications [23]. The best characterised mechanism of regulation of YAP is the phosphorylation of the Ser127 residue by LATS1 resulting in YAP cytoplasmic retention, but similar to what was shown in *Drosophila,* the regulation of YAP by phosphorylations seems to be much more complex [64,65]. This was evident in early studies that showed that the YAPS127A phospho-mutant had a different behaviour than the YAPS5A mutant which has mutations in the 5 putative LATS1/2 phosphorylation residues [4,64]. The work from Kawahara also indicated that several post-translational modifications regulate the interaction and pro-apoptotic signals of YAP-p73 downstream of LATS2 [78]. Moreover, Shaul’s group showed that the tyrosine kinase c-ABL promotes the binding of YAP-p73 by direct phosphorylation of YAPY357 upon DNA damage [134]. Importantly, this group also showed that this phosphorylation negatively regulates YAP-TEAD dependent transcription of pro-survival genes facilitating the activation of apoptosis [134]. Thus phosphorylation of YAPY357 seems to switch the transcriptional programs mediated by YAP. Similarly, Basu’s group showed that JNK regulates the pro-apoptotic signal mediated by YAP-p73 by direct phosphorylation of several residues including YAP Ser138, Ser317 and Thr362 [22]. The work from Lee *et al*. showed that the phosphorylation status changes upon genotoxic signalling and identified several residues that are phosphorylated by p38 and JNK preventing YAP-TEAD transcription [180]. YAP differential phosphorylation also regulates its protein level as shown by the work from Guan’s group indicating that phosphorylation of YAP in the Ser381 by LATS in the cytoplasm is necessary to allow the binding of CK1. This kinase in turn phosphorylates YAP in Ser384 and Ser387 [181]. These phosphorylations allow the binding of the E3 ubiquitin ligase SCF^β-TRCP^ to YAP, YAP ubiquitination by this protein and ultimately YAP degradation by the proteasome [181]. Therefore, it is clear that different kinases and phosphorylation residues regulate YAP dependent apoptosis. Interestingly, MST1 was recently shown to directly phosphorylate YAPS127 *in vitro* and in cells to regulate the interaction between YAP and androgen receptor in prostate cancer cell lines [182]. The existence of several kinases that regulate YAP functions was further illustrated during the study of the regulation of β-CAT by YAP. YAP seems to play a role in the regulation of β-CAT localisation and transcriptional activity. Hanh *et al.*, showed that YAP1 forms a transcriptional complex with β-CAT and the transcription factor TBX, that upon YES-catalysed phosphorylation of YAPY357, binds to the promotors of the anti-apoptotic genes *BIRC5* and *BCL2L1* [183]. Surprisingly, despite evidence of multiple regulatory phosphorylation sites of YAP in mammalian and *Drosophila* model, the phosphorylation status of the YAPS127 is still widely considered to be the crucial determinant of YAP activity. Frequently, the phosphorylation status of this residue is used as a read out for YAP “activation” in cancer, but this ignores the possibility that YAP may mediate its functions without changes in the phosphorylation levels of this residue. In fact, recent work from Pan’s group showing that *YAP*S112A (the mouse equivalent of human Ser127) knock-in mice are normal despite having increased nuclear YAP, which according to the authors, clearly caution against this assumption [177]. Moreover, YAP has recently been shown to work as a transcriptional co-repressor and, therefore, in some cases the lack of phosphorylation of YAP1 may be associated with inhibition of transcription [184]. This could also be the case for TEAD itself since it has been shown that this transcription factor also mediates the activation of a pro-apoptotic program that is prevented by its binding to YAP [185].

Further regulation of cell death by YAP may be dependent on non-transcriptional functions. The focus on YAP’s role as a co-transcription factor may be masking alternative functions of this protein in the cytoplasm such as its role as a regulator of β-CAT stability [183]. For instance, several works have shown that YAP and the E3 ligases NEED4 and ITCH compete for the binding to the PPxY sequence of different proteins of the pathway [186,187]. All these proteins contain a WW domain that binds to the PPxY sequence present in LATS1/2 and p63/p73 and probably the PPxF sequences of MST1/2 [187]. Through this mechanism, YAP has been shown to stabilise the protein levels of LATS1/2 and p73 [188,189]. Thus, YAP is able to regulate the protein levels of key nodes of the pathway, which is possibly related to the level of activation and outputs of the MST/Hippo signalling network. An example of the possible relevance of this mechanism was shown in epithelial cells treated with EGF where the levels of LATS1 are downregulated through increased ITCH expression, which seems to be necessary for the regulation of epithelial to mesenchymal transition of MDCK cells [190]. Additionally, several functions of YAP in the cytoplasm have been described. Some of these may be important in the regulation of cell death. An early example was the identification by Sudol that YAP may regulate cell polarity by localising to the apical membrane of epithelial cells. This localisation was shown to be regulated by YES phosphorylation, which promotes the binding of YAP to EBP50 [191]. Importantly, YAP has been proposed to prevent the activation of the WNT pathway by regulating β-CAT protein levels and localisation [16,192]. According to this model, YAP (and TAZ) binds to AXIN and is part of the β-CAT destruction complex. YAP therefore regulates the interaction of β-TrCP to the complex resulting in an inhibition of β-CAT translocation to the nucleus and regulation of target genes. The evidence for cytoplasmic functions of YAP is further supported by recent evidence showing that this protein is involved in the regulation of cytokinesis [193]. Thus, during mitosis YAP interacts with the polarity scaffold PATJ and YAP1 is necessary for the proper localisation of several proteins that regulate the contraction during cytokinesis including MgcRacGap, Anillin and RHOA [193]. Altogether, these observations clearly indicate that YAP is active in the cytoplasm.

In order to understand how YAP mediates the activation of cell death, knowing which splicing isoforms are expressed in the different cell types should also be taken into account when studying YAP functions [194]. To date 8 *YAP* transcripts have been predicted and several splicing isoforms have been shown to be expressed [194]. Importantly there are two major families of YAP isoforms, YAP1 and YAP2 that express one or two WW protein-protein interaction domains respectively. These two WW domains have been shown to be structurally different which possibly indicates the existence of different interactomes and functions for both isoforms [195]. The physiological relevance of YAP isoforms in cell death has been shown to be important in neuronal cells [196,197]. It has been shown in cell, animal and human models that neurons express a shorter YAP isoform called YAPΔC that differs from full length YAP because it lacks a region in the C-terminal region [197]. Different groups have shown that in neurons full length YAP triggers cell death by regulation of p73. Importantly, this cell death is not classical apoptosis but a very slow form of programmed cell death called TRIAD (transcriptional repression-induced atypical death) [197]. On the other hand, TRIAD seems to be prevented by YAPΔC which acts as dominant inhibitor of p73 dependent transcriptional activity. The balance between both YAP isoforms concentration seems to determine whether the cell initiates programmed cell death. Importantly the data from human clinical samples shows that the balance between both isoforms is modified in several neurodegenerative diseases, where the anti-cell death isoform YAPΔC is lost as the disease progresses which correlates with increased neuronal cell death [197]. Thus, better characterisation of YAP isoforms could help identifying redundant and non-redundant functions of these proteins and give us better understanding of YAP-dependent cell death.

In summary, the knowledge that we have about YAP biological functions is still very limited and we should not be blinkered by the assumption of canonical YAP signalling. YAP may be a potent oncogene in one context, but this should not interfere with its possible role in many other biological functions that have been proposed so far. These antagonistic functions of YAP probably depend on different stimuli, the cell type, the presence/absence of different partners, the existence of concomitant mutations and p53 status [14,19,178,198]. It is important to remember that other oncogenes have dual functions as tumour suppressors and oncogenes including KRAS and MYC [2]. Only the systematic study of YAP in relation to its signalling networks and different stimuli will help deciphering all the facets of this protein.

## 4. The MST/Hippo Pro-apoptotic Network in Human Disease

Given the complexity of the signalling networks that is mediated by the MST/Hippo pathway it is not surprising that the proteins of the pathway have been proposed to be involved in the development of several human pathologies. The discovery of its role in cancer has boosted the research of this network but it has also obscured the implication of the core proteins in other diseases such as diabetes, Alzheimer’s disease, ALS or cardiac failure.

### 4.1. Cancer

The role of the proteins in cancer has been extensively reviewed elsewhere [1,8,14,19,23]. The implication of the proteins of the canonical Hippo pathway in cancer seems to be clearly established and supported by abundant clinical data [7]. This has led to the general view that the core of the pathway is formed by a series of tumour suppressors that prevent the inappropriate activation of YAP which is an oncogene, and that its effect is mediated mainly by TEAD proteins. This view ignores the existence of other effectors of YAP and in particular of LATS1/2 [13,23]. Based on this view several groups and companies have started screening for drugs that can disrupt YAP-TEAD interaction with the hope that preventing the proliferative signal of this complex will result in tumour cell death and therefore function as an effective anticancer treatment [18]. These screenings have started giving results, and at least one drug, Verteporfin, has been shown to prevent cell proliferation by disrupting the YAP-TEAD complex and preventing its transcriptional activation [199,200]. Thus, the study of the pathway is becoming more translational and clinically relevant. However, based on what we know about targeting single nodes of signalling pathways for the treatment of cancer, we should be cautious about the possible lack of efficacy of these drugs especially if taken into account the evidence that the pathway belongs to a very complex signalling network. The best illustration comes from the use of different inhibitors of the proteins of the MAPK and AKT pathways [90,201,202]. Inhibitors developed against MEK or PI3K have been shown to have limited effects as first treatment agents in most tumours [202,203]. Similarly, specific mutant BRAF inhibitors that were shown to be extremely effective in the treatment of metastatic melanoma have a limited therapeutic window and most of the patients develop resistance to this treatment within a year [204]. In both cases, it is clear that the limited effect of these drugs is related to the capacity of cancer cells to rewire their signalling networks to avoid cell death. There is no reason to expect that the MST/Hippo signalling pathway and in particular, YAP would be an exception. In fact there is a possibility that YAP inhibitors not only have to inhibit YAP-TEAD transcription, but also have to allow the activation of YAP pro-apoptotic effectors such as p73 or EGR1 [179]. Moreover, it is possible that these inhibitors could prevent the interaction and activation of these pro-apoptotic effectors, which can have consequences for the response of the tumours to these treatments and preclude the possibility of undesired side effects in the patients.

The data included in this review illustrating the important role of the MST/Hippo network in the regulation of apoptosis must be also considered in the context of human tumours. Mutations of the members of the canonical pathway are rare including *YAP* of which, to the best of our knowledge, no activating mutation of the Ser127 has been described in human tumours [205]. YAP has been shown to be upregulated by gene duplication in several tumours but also loss of expression has been shown to correlate with worst prognosis at least in breast cancer [1,20]. This data indicates that this protein may have a dual role as oncogene and tumour suppressor depending on the tumour type. Additionally, MST1/2 and LATS1/2 have been shown to be downregulated by DNA methylation in several cancers [11,13]. Importantly, *RASSF1A* is one of the most frequently deregulated genes in solid tumours [102]. The common loss of expression of RASSF1A by DNA methylation is probably the most common event in cancer that prevents MST/Hippo dependent apoptosis in tumour cells. Additionally, other members of the *RASSF* family that regulate the pathway are also tumour suppressors, and are downregulated by DNA methylation [28]. Critically, the MST/Hippo network include some of the most commonly deregulated genes in human cancer such as p53, AKT, β-CAT, the RAF family and KRAS [14,16,66]. Therefore, the MST/Hippo network is highly deregulated in cancers and it will be necessary to get a complete understanding of the mechanisms that govern it in order to understand the contribution of the proteins of the MST/Hippo pathway in tumour development. Furthermore, while animal models are helpful to understand tumours they also have limitations. For example, in the case of the Hippo pathway, liver cancer is the most studied mouse model, where YAP overexpression and expression of the artificial mutant YAPS127 causes hyperplasia, which is reverted when this expression is decreased [4]. Other models have shown that loss of expression of MST1/2, SAV and RASSF1A result in the development of hepatic tumours [68,206]. While these models are useful, it is important to remember that 80% of liver cancer are caused by chronic infection with hepatitis virus B and C (HV) viruses [207]. The mechanisms by which HV lead to cancer disease include rewiring of signalling networks and induction of chronic inflammation [207]. Thus, the animal models have clear limitations and may not reflect the most common features of liver cancer development. A study of MST/Hippo pathway nodes that are commonly deregulated in hepatitis-induced cancer is likely to shed light on the role of this pathway in hepato-carcinoma. Interestingly, clinical and experimental data show that HV-C and B infection downregulate the expression of several tumour suppressors of this network including p53, p73, PTEN and RASSF1A [208]. In fact, it has been shown that the HV concomitantly downregulate p73 and RASSF1A which would result in a loss of YAP pro-apoptotic signal and probably activation of YAP-TEAD proliferative signal [209,210]. Concomitant targeting of RASSF1A and p73 was also shown in lung cancer where microRNA-602 reduced the expression of these members of the network and contributed to tumour development [209]. In light of this evidence, it is likely that further clinical studies where the whole network is taken into consideration, could result in the identification of new biomarkers and drug targets for several cancers.

### 4.2. Neurodegenerative Diseases

Neurodegenerative diseases are a group of diseases characterised by the death of neurons [211]. These diseases include among other Alzheimer’s, ALS and frontotemporal dementia, all of which have no effective treatment. The mechanisms that cause cell death are very poorly understood but it is thought that some of them are common to all of these diseases [211]. Prevention of neuronal cell death would stop the progression of these diseases. To a certain extent, these disorders are the opposite of cancer since they are caused by a loss of cells due to improper activation of cell death. As illustrated in this review, the proteins of the pathway have been shown to be involved in neuronal cell death in cell lines, animal models and patients samples [212]. Importantly, as with many pathologies, deregulation of signalling networks are thought to play an important role in the development of the disease. For example β-Amyloid is thought to prevent AKT pro-survival signal, and different strategies are being tested to amplify this signal as a possible treatment for Alzheimer’s [213]. As explained before, β-Amyloid seems to activate also the MST-FOXO pathway and it is possible that the loss of AKT direct inhibition of MST1 could explain this observation [158]. The observation of YAP-p73 dependent cell death in ALS is also important evidence illustrating the role of the proteins of the pathway in this disease [196]. The contribution of the MST/Hippo network in the nervous systems degeneration must be further confirmed but it is tempting to speculate that this may open the avenue for effective treatments of this fatal diseases.

### 4.3. Diabetes

The role of the pathway in neurodegenerative disease may have a close link to the possible involvement of MST1, LATS1 and other proteins of the pathway in the death of pancreatic β-cells that occurs in the development of diabetes [152,213]. According to the data that we have so far, it seems that this disease arises when the cells that produce insulin die; in this case, it seems to be by apoptosis. Importantly, MST1 would not only regulate the activation of apoptosis but it is also necessary for the desensitization of β-cells to glucose, the other hallmark of diabetes [152]. Again, deregulation of some other nodes in the network is thought to be common in diabetes and in particular insulin is a regulator of AKT and metabolism. The relation of the network with metabolism is only beginning to be deciphered but may be related to the role of the Hippo pathway in diabetes [214].

### 4.4. Cardiomyopathy

The death of myocytes after heart failure produce fibrosis that can severely affect heart function and for this reason the identification of methods and drugs that can prevent this cell death and promote myocyte proliferation is the object of intense research [165,215]. After heart infarction the MST1, LATS1/2 and YAP1 have been shown to play a role in the regulation of myocyte cell death [215,216]. Again, as in the case of diabetes and neurodegenerative diseases the aim would be to inhibit the pro-apoptotic signals mediated by the pathway and probably promote the proliferative signals triggered by other nodes.

In summary, the MST/Hippo signalling network may be important in the development of several diseases caused by aberrant deregulation of the balance between pro-survival and cell death signals. Even if the knowledge is still limited, the list of diseases described above have a high socio-economical effect. They include two of the most devastating epidemic diseases of the XXI century, diabetes and Alzheimer’s, and the three most common causes of death cancer, cardiovascular disease and neurological disease. The study of the common features of these diseases at the molecular level could help to identify some unexpected common mechanism between these diseases and hopefully will lead to the development of new treatments against some of these pathologies. In addition to these diseases, the wider MST/Hippo network has been proposed to be deregulated and involved in the development of other pathologies and the numbers are rapidly increasing. For example, it may play a role in intestinal disease (reviewed in [217]). It has also recently been shown that the MST/Hippo network may also be involved in fatty liver pathology since LATS2 regulates cholesterol accumulation in the liver [218].

## 5. Outlook

Since the description of the mammalian MST/Hippo pathway, our knowledge of the pathway has increased at an impressive rate. The combination of genetic models, classical cellular and molecular biological studies, the use transcriptomics and proteomics techniques along with clinical evidence, is showing that this pathway is at the cross-road of several signalling networks and regulates a plethora of biological functions that we are only beginning to comprehend. The data gathered in this first decade of the pathway, shows that the MST/Hippo proteins are a crucial part of the key signalling networks that regulate cellular homeostasis. As we unravel this complex network, we should expect surprises that expand the current view of the functions and regulations of this network. Understanding the functions of the core proteins of the pathway will help to complete our understanding of some of the molecular mechanisms regulating cell physiology and how best to tackle several pathologies. To do this we have to get a systemic understanding of this network and consider all the biological functions of the pathway that the data is pointing at, both varied and antagonistic.

## Abbreviations

The following abbreviations are used in this manuscript:

MAPK: Mitogen activated protein kinase
YAP: Yes-associated protein
LATS1/2: large tumour suppressor 1/2
MST1/2: mammalian sterile 20-like kinase ½
RASSF: Ras association domain family
SARAH: Salvador-RASSF-Hippo
TEAD: TEA domain family member
TAZ: Tafazzin
dSTRIPAK: *Drosophila* Striatin-interacting phosphatase and kinase WNT: wingless-type MMTV site family
SHH: sonic hedgehog
TGF: transforming growth factor
AKT: v-akt murine thymoma viral oncogene homolog
Ras: rat sarcoma virus oncogene
RAF: Rat fibrosarcoma
PI3K: phosphatidylinositol-4,5-bisphosphate 3-kinase
